# Risk factors for gastric cancer: A comprehensive analysis of observational studies

**DOI:** 10.3389/fpubh.2022.892468

**Published:** 2023-01-04

**Authors:** Yuqing Hui, Chunyi Tu, Danlei Liu, Huijie Zhang, Xiaobing Gong

**Affiliations:** Department of Gastroenterology, The First Affiliated Hospital, Jinan University, Guangzhou, Guangdong, China

**Keywords:** gastric cancer, risk factors, protective factors, comprehensive analysis, quality

## Abstract

**Background:**

Multifarious factors have a causal relationship with gastric cancer (GC) development. We conducted a comprehensive analysis to evaluate the strength of the evidence examining non-genetic risk factors for gastric cancer.

**Methods:**

PubMed, Web of Science, and the Cochrane Library were searched from inception to November 10, 2021 to identify meta-analyses of observational studies examining the association between environmental factors and GC risk. For each meta-analysis, the random effect size, 95% confidence interval, heterogeneity among studies, and evidence of publication bias were assessed; moreover, the evidence was graded using predefined criteria, and the methodological quality was evaluated using AMSTAR 2.

**Results:**

A total of 137 associations were examined in 76 articles. Among these meta-analyses, 93 associations yielded significant estimates (*p* < 0.05). Only 10 associations had strong epidemiologic evidence, including 2 risk factors (waist circumference and bacon), and 8 protective factors (dietary total antioxidant capacity, vegetable fat, cruciferous vegetable, cabbage, total vitamin, vitamin A, vitamin C, and years of fertility); 26 associations had moderate quality of evidence; and the remaining 57 associations were rated as weak. Ninety-four (68.61%) associations showed significant heterogeneity. Twenty-five (18.25%) associations demonstrated publication bias.

**Conclusions:**

In this comprehensive analysis, multiple associations were found between environmental factors and GC with varying levels of evidence. Healthy dietary habits and lifestyle patterns could reduce the risk for GC. However, further high-quality prospective studies are still necessary to draw more definitive conclusions.

## Background

The incidence of gastric cancer (GC) has gradually decreased in recent decades, mainly due to improved socioeconomic status, hygienic practices, and consequentially reduced Helicobacter pylori (HP) infection rates (1–3). However, GC remains the fifth most common cancer and the third major cause of oncological mortality worldwide (4, 5), and is responsible for over 1,000,000 new cases and approximately 80,000 deaths per year (4), which has posed a serious global public health burden. The etiology of GC is complicated and multifactorial; both genetic and environmental risk factors together with their interaction significantly contribute to its development (6, 7). A better understanding of these risk factors may improve the prediction and prevention of this condition.

Although many systematic reviews and meta-analyses have examined risk factors for GC (8), to our knowledge, there have been no systematic efforts to summarize and critically evaluate the evidence. Therefore, the aim of this comprehensive analysis is to provide a comprehensive overview and assess the strength, credibility, and classification of the existing epidemiological evidence examining the association between non-genetic factors and GC risk (9).

## Methods

This study was registered in the International Prospective Register of Systematic Reviews (registration number: CRD42021290515). This study did not require ethical approval.

### Literature search and eligible criteria

Two observers (HYQ and TCY) independently and systematically searched PubMed, Web of Science, Cochrane Library from inception to November 10, 2021, to identify observational studies of systematic reviews and meta-analyses assessing the association between non-genetic risk factors and GC using the following search algorithm: (“Stomach Neoplasms” OR “Gastric Cancer” OR “Cancer, Gastric” OR “Cancers, Gastric” OR “Gastric Cancers” OR “Gastric Neoplasm” OR “Gastric Neoplasms” OR “Stomach Neoplasm” OR “Neoplasm, Stomach” OR “Neoplasms, Stomach” OR “Neoplasms, Gastric” OR “Neoplasm, Gastric” OR “Stomach Cancers” OR “Cancers, Stomach” OR “Cancer, Stomach” OR “Stomach Cancer” OR “Cancer of the Stomach”) AND (“systematic review” OR “meta-analysis”). The reference lists of retrieved eligible papers were further hand-searched to avoid missing other potentially related studies. Only articles published in English were included.

Articles were deemed qualified if they satisfied all of the following inclusion criteria: (1) the articles were systematic reviews and meta-analyses of observational studies (i.e., cohort and case-control and cross-sectional studies); (2) the study evaluated the association of environmental (non-genetic) factors and GC risk, but not for screening, diagnostic, prognostic purposes; and (3) the study provided enough data to perform the analyses. The title and abstract of all eligible papers were initially screened, and then the full text of possible qualified articles was retrieved for further perusal based on the pre-established inclusion and exclusion criteria. Disagreements between two investigators were settled through a discussion. If multiple meta-analysis examined the identical scientific issue, we chose the largest number of studies to avoid repeated evaluation of the same primary studies (10, 11).

### Data extraction

From each included meta-analysis, two researchers (HYQ and TCY) independently extracted the following data: the examined risk factors, the first author's name, year of publication, the epidemiological design and number of included studies, and the number of participants and cases. The study-specific relative risk estimates [i.e., relative risks (RRs), odds ratios (ORs), and hazard ratios (HRs) together with their corresponding 95% confidence intervals (CI)], heterogeneity, and publication bias for every risk factor were also collected in each study. Divergence during data extraction was clarified by discussion until a consensus was reached.

### Assessment of methodological quality

Two researchers (HYQ and TCY) independently appraised the methodological quality of all included systematic reviews using the updated 16-item AMSTAR 2 instrument (a measurement tool for assessing the methodological quality of systematic reviews) (12). This tool is used to classify the methodological quality into four grades: high, moderate, low, and critically low. No or only one non-critical flaw is considered high quality, more than one non-critical defect is considered moderate quality, only one critical defect with or without non-critical flaws is low quality, and more than one critical defect with or without non-critical flaws is considered critically low quality. Any differences between the AMSTARS 2 scores were resolved through a discussion or arbitration by the third investigator (LDL).

### Evaluation of the evidence quality

Two authors (HYQ and TCY) independently assessed the strength of the epidemiologic evidence using the following criteria (11, 13, 14):

(1) precision of the estimate (*p*-value <0.001 (15, 16), a threshold related to significantly fewer false-positive results, and >1,000 cases), (2) the heterogeneity between studies was not relatively large (I^2^ <50% and *p*-value of Cochran Q test>0.10), and (3) no evidence of small-study effects (*p*-value of Egger's test>0.10). The strength of the epidemiologic evidence was classified as high (when all of these criteria were met), moderate (when a maximum of 1 criterion was not met and *p*-value < 0. 001 was satisfied), or weak (in other cases, *p*-value < 0.05). When the *p*-value was not directly reported, it was recalculated from the 95% confidence interval of the pooled effect estimate using an established method (17). In case of doubt during the evaluation of evidence quality, discrepancies were settled through arbitration with a third investigator (LDL).

### Data synthesis and analysis

Based on the extracted raw data from every included study, we reanalyzed and presented the random-effects estimate whenever the fixed-effects model was initially used (14). And in the case of missing measures, we calculated them when enough data were available. A *p*-value of the pooled estimate of effect size of < 0.05 was deemed significant. I^2^ and Q test was used to determine the heterogeneity (18) among studies, while Egger's test was utilized to evaluate the small-study effects (19); a *p*-value of < 0.10 (Q test) indicated a significant heterogeneity and a publication bias. Values (I^2^ test) exceeding 50% were generally considered to indicate high heterogeneity. All *p*-values were two tailed, and all statistical analyses were performed using Stata version 16.0.

## Results

### Characteristics of the included studies

**Figure 1:**
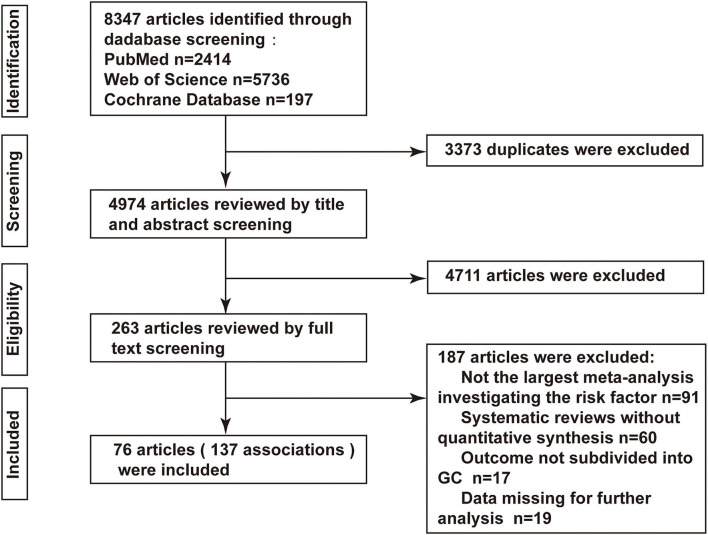
Flow diagram for literature search and selection process.

Overall, the initial search identified a total of 8,347 articles (2,414 from PubMed, 5,736 from Web of Science, and 197 from the Cochrane Library), of which 76 were finally deemed eligible (20–95). The process of selecting contained meta-analyses is displayed in Figure 1, while the general characteristics of the included eligible articles are summarized in Supplementary Table S1. The publication dates of contained studies ranged from 2008 to 2021. Among the meta-analyses reported in our study, the median number of original articles included in each meta-analysis was 12 (range: 2–73), the median number of cases was 4,745 (range: 51–137,451), and the number of cases was >1,000 in 113 (82.48%) meta-analyses. All 76 articles examined 112 unique risk factors and 137 associations, among these associations, 93 (67.88%) reported significant summary effects with a *p*-value of <0.05, while 47 (34.3%) reported a *p*-value of <0.001 (Supplementary Table S1).

### Anthropometric indices

**Figure 2:**
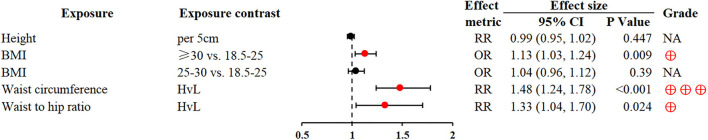
Forest plot: summary effect estimates of meta-analyses reporting associations between GC and factors pertaining to anthropometric indices. BMI, body mass index; HvL, highest vs. lowest; NA, not applicable; red dots represent risk factors; blue dots represent protective factors; The strength of the epidemiologic evidence was rated as high (⊕⊕⊕), moderate (⊕⊕), weak (⊕).

Obesity is a well-recognized risk factor for multiple adverse health outcomes. Waist circumference and waist–to–hip ratio were associated with an increased risk of GC in the highest vs. lowest comparisons (RR 1.48; 1.24–1.78 and RR 1.33; 1.04–1.70, respectively) (20). Similarly, a higher body mass index (≥30 vs. 18.5-25) was also associated with GC (OR 1.13; 1.03–1.24) (22) (Figure 2).

### Dietary intake

**Figure 3:**
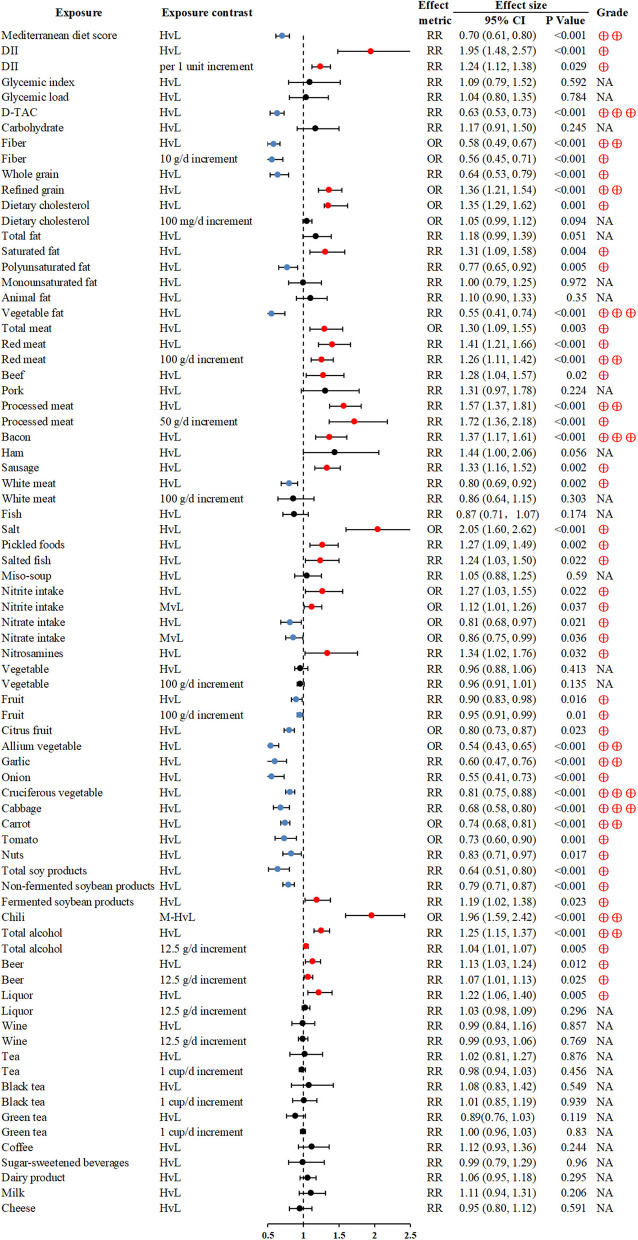
Forest plot: summary effect estimates of meta-analyses reporting associations between GC and factors pertaining to dietary intake. DII, dietary inflammatory index; D-TAC, dietary total antioxidant capacity.

The Mediterranean diet (MedDiet) and dietary total antioxidant capacity (D-TAC) were associated with significant reductions in GC risk for the highest vs. lowest comparisons (RR 0.7; 0.61–0.8 and RR 0.63; 0.53–0.73; respectively) (23, 26). Fiber intake was inversely associated with GC (28), whereas, refined grain consumption of the highest dosage was related to a significantly increased risk of GC (OR 1.36; 1.21–1.54) (30). Intake of meat, particularly red and processed meat was associated with GC (34, 94). High salt consumption, especially salt-preserved foods, could increase the GC risk (36, 37). Fruit and vegetable intake were widely reported as protective factors for GC (40, 41, 43); of note, vegetable fat also had beneficial effects on GC (RR 0.55; 0.41–0.74) (32). Heavy alcohol drinking and chili intake might increase the risk of GC (48, 49), but no association between tea and GC was found (50) (Figure 3).

### Micronutrients

**Figure 4:**
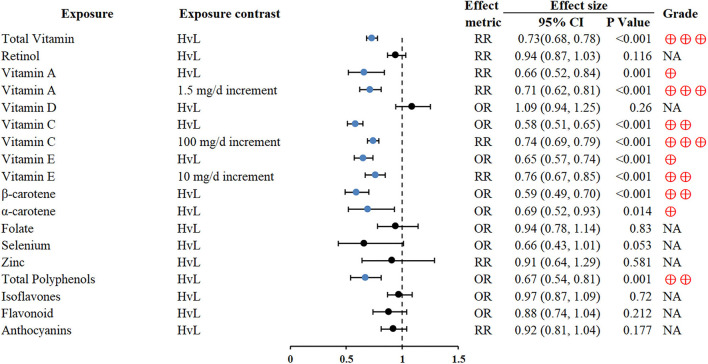
Forest plot: summary effect estimates of meta-analyses reporting associations between GC and factors pertaining to micronutrients.

Higher vitamin consumption was associated with reduced GC risk (RR 0.73; 0.68–0.78) (55), especially antioxidant vitamins (vitamin A, vitamin C, vitamin E, β-carotene, and α-carotene). An inverse association between total polyphenol intake and GC was also found (OR 0.67; 0.54–0.81, for the highest vs. lowest intake comparisons) (62) (Figure 4).

### Use of medication therapy

**Figure 5:**
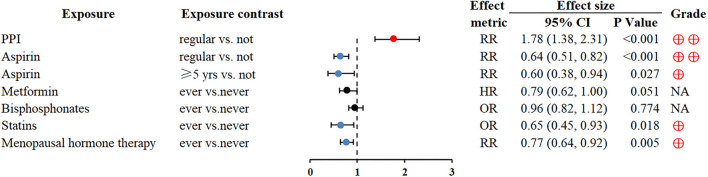
Forest plot: summary effect estimates of meta-analyses reporting associations between GC and factors pertaining to use of medication. PPI, proton pump inhibitors.

Regular proton pump inhibitors (PPI) use was associated with GC (RR 1.78; 1.38–2.31) (66). Conversely, regular aspirin use could reduce the risk of GC (RR 0.6; 0.51–0.82) (67). In addition, statin use and menopausal hormone therapy might also be related to a decreased risk of GC (OR 0.65; 0.45–0.93 and RR 0.77; 0.64–0.92, respectively) (71, 72) (Figure 5).

### Lifestyle

**Figure 6:**
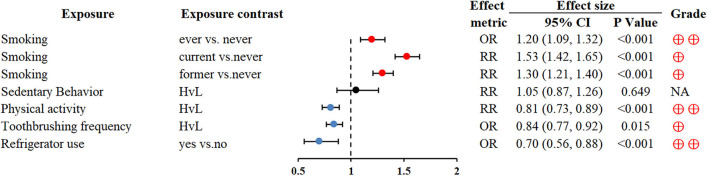
Forest plot: summary effect estimates of meta-analyses reporting associations between GC and factors pertaining to use of lifestyle.

Smoking can be linked to the development of many cancers, including GC (73, 74). Physically active people were protected from subsequent GC (RR 0.81; 0.73–0.89) (76). Higher toothbrushing frequency and refrigerator use also reduced the risk of GC (OR 0.84; 0.77–0.92 and OR 0.7; 0.56–0.88, respectively) (77, 78) (Figure 6).

### Pre-existing medical history

**Figure 7:**
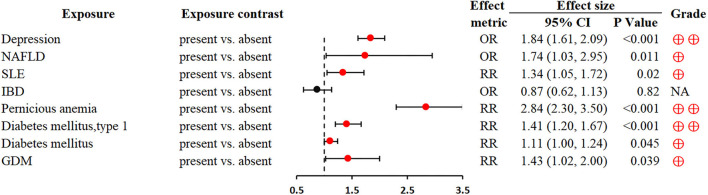
Forest plot: summary effect estimates of meta-analyses reporting associations between GC and factors pertaining to pre-existing medical history. NAFLD, non-alcoholic fatty liver disease; SLE, systemic lupus erythematosus; IBD, inflammatory bowel disease; GDM, gestational diabetes mellitus.

Depression was associated with an increased GC risk (OR 1.84; 1.61–2.09) (79). Non-alcoholic fatty liver disease (NAFLD) could significantly increase the development risk of various extrahepatic cancers, including GC (OR 1.74; 1.03–2.95) (80). Based on the contribution of autoimmunity to gastric carcinogenesis, autoimmune diseases (e.g., systemic lupus erythematosus, pernicious anemia and diabetes mellitus, type 1) were closely associated with an increased risk of GC (RR 1.34; 1.05–1.72; RR 2.84; 2.30–3.50; RR 1.41; 1.2–1.67, respectively) (81, 83) (Figure 7).

### Viral or bacterial infection

**Figure 8:**
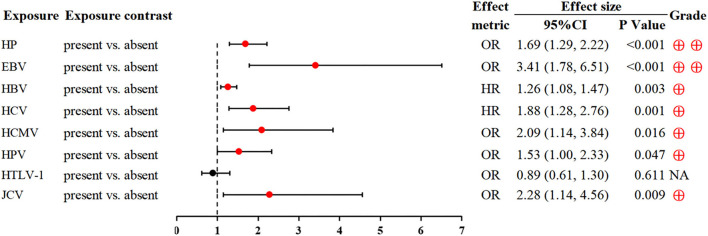
Forest plot: summary effect estimates of meta-analyses reporting associations between GC and factors pertaining to viral or bacterial infection. HP, Helicobacter pylori; EBV, Epstein-Barr virus; HBV, hepatitis B virus; HCV, hepatitis C virus; HCMV, human cytomegalovirus; HPV, human papillomavirus; HTLV-1, human T-cell lymphotropic virus type 1; JCV, John Cunningham virus.

In addition to well-recognized HP and Epstein-Barr virus, other viruses (e.g., hepatitis B virus, hepatitis C virus, human cytomegalovirus, human papillomavirus and John Cunningham virus) were also associated with a higher incidence of GC (86–89) (Figure 8).

### Other factors

**Figure 9:**
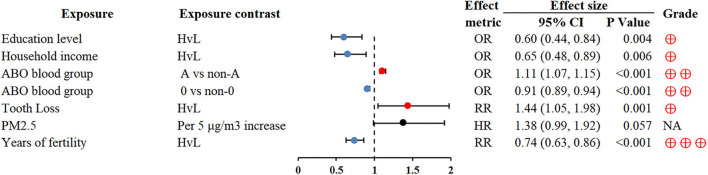
Forest plot: summary effect estimates of meta-analyses reporting associations between GC and factors pertaining to other factors. PM2.5, particulate matter with a diameter of 2.5 μm or less.

A strong inverse association was found between socioeconomic position indicators (educational level and household income) and GC risk (OR 0.60; 0.44–0.84 and OR 0.65; 0.48–0.89, respectively) (90). Of note, we found that blood group O and longer duration of fertility were protective factors of GC, conversely, blood group A was associated with a higher GC risk (72, 91) (Figure 9).

### Heterogeneity and small study effects

With regard to heterogeneity, in the 137 unique meta-analyses, only 43 (31.39%) associations showed absence of heterogeneity (I^2^ < 50% and *p-*value of Cochran Q test ≥ 0.10), while the remaining 94 (68.61%) associations indicated significant heterogeneity (I^2^ ≥ 50% or *p*-value of Cochran Q test < 0.10). In terms of publication bias, 110 (80.29%) meta-analyses showed no evidence of significant small-study effects (*p*-value ≥ 0.10 of Egger's test), whereas 25 (18.25%) meta-analyses demonstrated publication bias (*p*-value < 0.10 of Egger's test). With regard to the associations of GC with fiber intake (10 g/d increment) (28) and HTLV-1 infection (89), small-study effects were not applicable as only 2 observational studies were included in each meta-analysis.

### Quality assessment of meta-analyses

**Figure 10:**
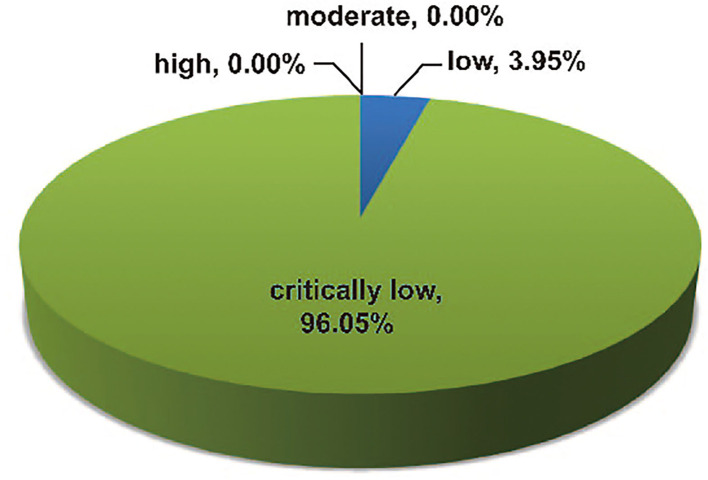
Map of results of AMSTAR 2 scores.

The methodological qualities of the 76 included articles were assessed and graded using the 16-item AMSTAR2 tool (Supplementary Table S2); three (3.95%) studies were determined to have low methodological quality, while the other 73 (96.05%) studies were determined to have critically low methodological quality (Figure 10). Based on the AMSTAR 2 scores, none of the eligible articles had high or moderate methodological quality. The most common critical flaws were as follows: lack of registered protocol (*n* = 64, 84.21%), incomplete literature search (*n* = 75, 98.68%), and the absence of list of excluded studies (*n* = 64, 84.21%).

### Strength of epidemiologic evidence

**Figure 11:**
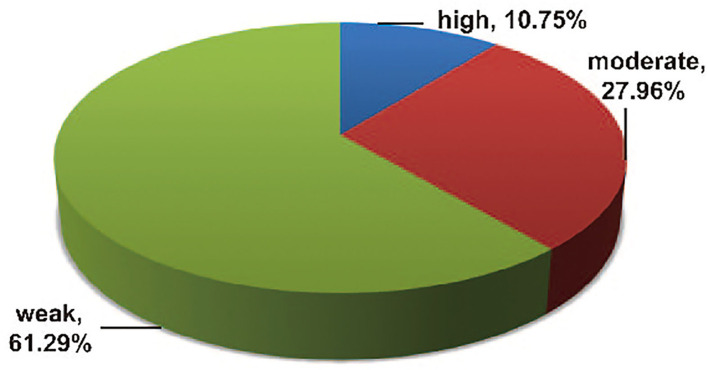
Map of results of epidemiologic evidence assessment.

The outcomes of the epidemiologic evidence measurement are presented in Supplementary Table S3. Among the 93 statistically significant associations, only 10 (10.75%) showed high epidemiologic evidence for an association with GC according to the abovementioned prespecified credibility criteria (with >1,000 cases, *p*-value of < 0.001, and absence of large heterogeneity and small-study effects), including two risk factors (waist circumference and bacon) and eight protective factors (D-TAC, vegetable fat, cruciferous vegetable, cabbage, total vitamin, vitamin A [HvL], vitamin C [100 mg/d increment], and years of fertility) (20, 26, 32, 43, 55, 56, 72, 94). A total of 26 (27.96%) associations demonstrated moderate epidemiologic evidence, the remaining 57 (61.29%) associations presented weak epidemiological evidence (Figure 11).

## Discussion

In this comprehensive analysis, 76 meta-analyses of observational studies were identified and appraisal of current evidence that examined the association of GC with various environmental risk factors was performed. All 137 environmental associations, covering anthropometric indices, dietary intake, micronutrients, use of medication, lifestyle, pre-existing medical history, viral or bacterial infection, and others, were assessed. Among these, two risk factors (waist circumference and bacon) and eight protective factors (D-TAC, vegetable fat, cruciferous vegetable, cabbage, total vitamin, vitamin A, vitamin C, and years of fertility) yielded high epidemiologic evidence without any evidence of bias (20, 26, 32, 43, 55, 56, 72, 94). However, we cannot confirm if other connections are not meaningful, and some uncertainties still need to be evaluated further.

The association between waist circumference and increased risk of GC was supported by high epidemiologic evidence (20), indicating that abdominal obesity plays a major role in the development of GC, and this is consistent with previous studies (96–98). The potential molecular mechanism of carcinogenesis is as follows: the metabolically active visceral adipose tissues promote the production of inflammatory mediators and cytokines (e.g., TNF-α and leptin), inhibit the secretion of adiponectin, and facilitate the development of insulin resistance (99, 100) and subsequent hyperinsulinemia to partially promote carcinogenesis by stimulating the increase in the expression of insulin-like growth factor (IGF-1) (101).

Of note, more than half of the associations examined a broad variety of dietary factors, which revealed the current direction of this line of research. The MedDiet is a recognized healthy dietary pattern, characterized by relatively high consumption of fruits, vegetables, legumes, and olive oil, moderate intake of dairy products and fish, and very limited intake of red meat and processed meat products (23, 102). Our results showed that the MedDiet is inversely associated with GC risk by 30% (23). Fruits and vegetables are rich sources of dietary fiber and antioxidant vitamins. Higher consumption of dietary fiber increases stool bulk, thereby diluting and slowing the absorption of potential carcinogens (103). Fiber can also be fermented into short-chain fatty acids by gut microbiota to exert antitumor effects (28). That is why a higher intake of refined grains increases the risk of GC (30). Antioxidant vitamins can scavenge free radicals, enhance antioxidative capacity and reduce cell oxidative damage (104). Potential carcinogens (e.g., N-nitroso compounds, polycyclic aromatic hydrocarbons and heterocyclic aromatic amines) and lipid peroxidation may explain the positive association between excessive red and processed meat intake and GC (34). Pickled foods, as a potential source of nitrosamines, are associated with a higher GC risk (37). Heavy alcohol drinking is closely related to GC, mainly due to the oxidative stress and DNA damage induced by its metabolite, acetaldehyde, and the direct damaging effect of ethanol on gastric mucosa (49).

With regard to medication therapy, long-term use of PPIs could increase GC risk, as supported by moderate epidemiologic evidence, mainly due to the accelerating progression of HP-related atrophic gastritis- and hypergastrinemia-promoting enterochromaffin-like cell hyperplasia (105). However, based on our findings, aspirin showed moderate epidemiologic evidence of GC- preventive effects, likely owing to the inhibition of cyclooxygenase-2 (COX-2) (67). In terms of pre-existing medical conditions, depression, pernicious anemia, and type 1 diabetes mellitus presented an increased risk of GC with moderate credibility. Depression can degrade the immune and endocrine systems, thus reducing resistance to cancer (106). Depression can also promote the secretion of glucocorticoids due to the influence of the hypothalamus–pituitary–adrenal axis, causing gastric mucosal erosion and ulcers (107). The positive association between pernicious anemia and GC is mainly due to the destruction of acid-producing parietal cells (108).

The estimated 60 well-established carcinogens found in cigarette smoke could explain the positive association between smoking and GC risk (109). Moderate credibility indicated that the risk of GC was increased in individuals with blood group A, but was significantly reduced in individuals with blood group O. Individuals with blood group A is more susceptible to GC partly due to the reduced immune system's response to tumors and the increased risk of pernicious anemia (110) and HP infection (111). The longer duration of fertility showed highly credible evidence of an inverse association with GC, mainly owing to the effect of estrogen (72).

Most of the assessed associations could not show high epidemiological evidence due to the significant heterogeneity and/or small-study effects. Heterogeneity usually indicates the presence of bias in some primary studies, but might also be due to the real differences among studies. Genuine heterogeneity might play a role in the field of GC, including the difference in exposure assessment, the mixture of cohort and case-control studies in some meta-analyses, differential association of risk factors due to geographical heterogeneity, and so on. As positive results are more likely to be published compared with null results, and the study participants may be a small portion of the actual population with the disease, the probability of small-study effects should be taken into consideration. The reported associations with GC need to be explained with caution, particularly in meta-analyses with a relatively small number of included studies; the heterogeneity and publication bias among researches are evident.

## Strengths and limitations

Our study has several strengths. This comprehensive analysis was the first to provide a comprehensive overview of the evidence to evaluate the association of non-genetic factors with GC risk, although several studies reported the risk factors of GC. Then, the systematic literature search, article selection, and data extraction were conducted by two independent researchers. Additionally, the AMSTAR 2 tool was utilized to appraise the methodological quality of the eligible systematic reviews. Furthermore, the epidemiologic evidence was graded in accordance with the predefined criteria including the evaluation of the estimate precision, heterogeneity, and publication bias.

Nevertheless, our study has several limitations. First, only published meta-analyses of observational researches were included; thus, other risk factors with enough evidence that have not yet been evaluated by meta-analytic quantitative synthesis were possibly overlooked. Second, the quality of component meta-analyses was not evaluated as it exceeded the range reported in our study, and meta-analyses should be conducted by the researchers of the primary studies. Third, the majority of included meta-analyses showed heterogeneity, and the observational investigations were prone to selection and recall biases, especially case-control studies. Fourth, the systematic reviews and meta-analyses contained in our study were only published in English; thus, the possible missing information that were published in other languages might affect the evaluation results.

## Conclusions

In conclusion, developing a healthier dietary (e.g., MedDiet) and lifestyle pattern along with promoting physical activity to prevent obesity could hopefully reduce the incidence of GC in the near future. However, further high-quality prospective studies excluding potential residual confounders are needed; the application of reporting guidelines (e.g., STROBE) (112) and registration of hypothesis-testing observational studies may be necessary to improve the credibility of evidence. Updated methodologically robust meta-analyses are also needed to better understand the association of GC with these factors and draw firmer conclusions.

## Data availability statement

The original contributions presented in the study are included in the article/Supplementary material, further inquiries can be directed to the corresponding author.

## Author contributions

YH and XG contributed to the concept and design of the comprehensive analysis. YH, CT, and DL collected and analyzed the data. YH and HZ drafted the manuscript. XG revised the manuscript. All authors read and approved the final manuscript to be published.
