# Corrigendum: Risk factors for gastric cancer: A comprehensive analysis of observational studies

**DOI:** 10.3389/fpubh.2023.1172663

**Published:** 2023-03-24

**Authors:** Yuqing Hui, Chunyi Tu, Danlei Liu, Huijie Zhang, Xiaobing Gong

**Affiliations:** Department of Gastroenterology, The First Affiliated Hospital, Jinan University, Guangzhou, Guangdong, China

**Keywords:** gastric cancer, risk factors, protective factors, comprehensive analysis, quality

In the published article, there was an error in the author list. The contribution statement was erroneously excluded. The corrected author list appears below.

[Yuqing Hui^†^, Chunyi Tu^†^, Danlei Liu^†^, Huijie Zhang and Xiaobing Gong^*^^†^These authors have contributed equally to this work].

The authors apologize for this error and state that this does not change the scientific conclusions of the article in any way. The original article has been updated.

