# Supplementary material for: Risk factors for gastric cancer: A comprehensive analysis of observational studies
Source: Front Public Health. 2023 Jan 4;10:892468. doi: 10.3389/fpubh.2022.892468 (PMC9845896; doi:10.3389/fpubh.2022.892468)
Supplement: Supplementary file 1 [file Table_1.DOCX]

**Supplementary Table1.**

**Characteristics of 137 meta-analyses investigating environmental risk factors associations with GC.**

| **Exposure** | Exposure contrast | **Author,year** | **Cohort/Case-control /Total Studies(n)** | **Participants(n)** | **Case(n)** | **Type of metric** | **Effect size** | | **Heterogeneity** | | **Small-study effect** |
| --- | --- | --- | --- | --- | --- | --- | --- | --- | --- | --- | --- |
|  |  |  |  |  |  |  | **95% CI** | **P Value** | I**^2^** | **P Value** |  |
| **Anthropometric indices** |  |  |  |  |  |  |  |  |  |  |  |
| Height | per 5cm | Min Seok Seo,2020 | 7/4/11 | 30,787,072 | 137,451 | RR | 0.99 (0.95, 1.02) | 0.447^*^ | 64.3 | 0.05^*^ | 0.276 |
| BMI | ≥30 vs. 18.5-25 | Xue-Jun Lin,2014 | 11/3/14 | 5,174,663 | 26,508 | OR | 1.13 (1.03, 1.24) | 0.009^*^ | 7.7 | 0.368 | 0.52 |
| BMI | 25-30 vs. 18.5-25 | Xue-Jun Lin,2014 | 12/3/15 | 4,882,781 | 25,686 | OR | 1.04 (0.96, 1.12) | 0.39^*^ | 45.8 | 0.027 | 0.08 |
| Waist circumference | HvL | Xuan Du,2017 | 5/0/5 | 844,508 | 1,701 | RR | 1.48 (1.24, 1.78) | <0.001^*^ | 0 | 0.682 | 0.9^*^ |
| Waist to hip ratio | HvL | Xuan Du,2017 | 5/0/5 | 719,858 | 1,580 | RR | 1.33 (1.04, 1.70) | 0.024^*^ | 35.9 | 0.167 | 0.378^*^ |
| **Dietary intake** |  |  |  |  |  |  |  |  |  |  |  |
| Mediterranean diet score | HvL | Jakub Morze,2021 | 4/3/7 | NA | 3,860 | RR | 0.70 (0.61, 0.80) | <0.001 | 52 | 0.045^*^ | 0.515^*^ |
| DII | HvL | Ying Liang,2019 | 0/3/3 | 2118 | 700 | RR | 1.95 (1.48, 2.57) | <0.001^*^ | 42.7 | 0.174 | 0.046^*^ |
| DII | per 1 unit increment | Ying Liang,2019 | 1/2/3 | 101,835 | 475 | RR | 1.24 (1.12, 1.38) | 0.029^*^ | 84.3 | 0.002 | 0.367^*^ |
| Glycemic index | HvL | Federica Turati,2019 | NA/NA/7 | NA | 3,152 | RR | 1.09 (0.79, 1.52) | 0.592^*^ | 80.3 | <0.001 | 0.683^*^ |
| Glycemic load | HvL | Federica Turati,2019 | NA/NA/7 | NA | 3,152 | RR | 1.04 (0.80, 1.35) | 0.784^*^ | 48.1 | 0.061 | 0.948^*^ |
| D-TAC | HvL | MohammadParohan,2019 | 2/2/4 | 485,608 | 1,179 | RR | 0.63 (0.53, 0.73) | <0.001 | 0 | 0.812 | >0.10 |
| Carbohydrate | HvL | Yao Ye,2017 | 2/20/22 | NA | NA | RR | 1.17 (0.91, 1.50) | 0.245 | 79.1 | <0.001 | 0.784 |
| Fiber | HvL | Zhizhong Zhang,2013 | 2/19/21 | 580,064 | 6,951 | OR | 0.58 (0.49, 0.67) | <0.001 | 62.2 | <0.001 | 0.931 |
| Fiber | 10 g/d increment | Zhizhong Zhang,2013 | 0/2/2 | 2045 | 478 | OR | 0.56 (0.45, 0.71) | <0.001 | 0 | 0.941 | NA |
| Whole grain | HvL | Xiao-Feng Zhang,2020 | 2/9/11 | 1,021,955 | 8,274 | RR | 0.64 (0.53, 0.79) | <0.001 | 78.2 | <0.001^*^ | 0.009^*^ |
| Refined grain | HvL | Tonghua Wang,2020 | 1/15/16 | 17,927 | 5,869 | OR | 1.36 (1.21, 1.54) | <0.001 | 56.5 | 0.002 | 0.564^*^ |
| Dietary cholesterol | HvL | Peng Miao,2021 | 0/14/14 | 19,989 | 5,174 | OR | 1.35 (1.29, 1.62) | 0.001 | 70.1 | <0.001 | 0.828 |
| Dietary cholesterol | 100 mg/d increment | Peng Miao,2021 | 0/8/8 | 26,433 | 6,692 | OR | 1.05 (0.99, 1.12) | 0.094^*^ | 84 | <0.001 | 0.18 |
| Total fat | HvL | Jun Han,2015 | 2/26/28 | 522,258 | 8,381 | RR | 1.18 (0.99, 1.39) | 0.051^*^ | 69.5 | <0.001 | 0.428 |
| Saturated fat | HvL | Jun Han,2015 | 2/16/18 | 509,749 | 4,906 | RR | 1.31 (1.09, 1.58) | 0.004^*^ | 60.6 | <0.001 | 0.15 |
| Polyunsaturated fat | HvL | Jun Han,2015 | 2/14/16 | 509,925 | 4,911 | RR | 0.77 (0.65, 0.92) | 0.005^*^ | 56.2 | 0.003 | 0.11 |
| Monounsaturated fat | HvL | Jun Han,2015 | 2/12/14 | 508,329 | 4,002 | RR | 1.00 (0.79, 1.25) | 0.972^*^ | 63 | <0.001 | 0.67 |
| Animal fat | HvL | Jun Han,2015 | 0/6/6 | 6,742 | 2,888 | RR | 1.10 (0.90, 1.33) | 0.35^*^ | 42 | 0.125 | 0.991^*^ |
| Vegetable fat | HvL | Jun Han,2015 | 0/4/4 | 4,167 | 1,764 | RR | 0.55 (0.41, 0.74) | <0.001^*^ | 48.6 | 0.12 | 0.96^*^ |
| Total meat | HvL | Ana Ferro,2019 | 0/22/22 | 39,472 | 11,443 | OR | 1.30 (1.09, 1.55) | 0.003^*^ | 77.9 | <0.001^*^ | 0.292^*^ |
| Red meat | HvL | Seong Rae Kim,2019 | 5/9/14 | 1,243,336 | 9,726 | RR | 1.41 (1.21, 1.66) | <0.001^*^ | 69.6 | <0.001 | 0.076 |
| Red meat | 100 g/d increment | Seong Rae Kim,2019 | 4/14/18 | 1,221,462 | 7,852 | RR | 1.26 (1.11, 1.42) | <0.001^*^ | 70.3 | <0.001 | 0.315 |
| Beef | HvL | Hongcheng Zhu,2013 | 1/7/8 | 152,948 | 2,625 | RR | 1.28 (1.04, 1.57) | 0.02^*^ | 0 | 0.47 | 0.849 |
| Pork | HvL | Hongcheng Zhu,2013 | 1/4/5 | 149,336 | 1,968 | RR | 1.31 (0.97, 1.78) | 0.224^*^ | 28.9 | 0.162 | 0.643^*^ |
| Processed meat | HvL | Seong Rae Kim,2019 | 10/23/33 | 1,326,493 | 10,645 | RR | 1.57 (1.37, 1.81) | <0.001^*^ | 55.5 | <0.001 | 0.448 |
| Processed meat | 50 g/d increment | Seong Rae Kim,2019 | 7/12/19 | 1,266,661 | 5,952 | RR | 1.72 (1.36, 2.18) | <0.001^*^ | 72.1 | <0.001 | 0.039 |
| Bacon | HvL | Hongcheng Zhu,2013 | 3/4/7 | 84,937 | 1,641 | RR | 1.37 (1.17, 1.61) | <0.001^*^ | 0 | 0.659 | 0.512 |
| Ham | HvL | Hongcheng Zhu,2013 | 2/3/5 | 66,231 | 1,134 | RR | 1.44 (1.00, 2.06) | 0.056^*^ | 77.9 | 0.001 | 0.314 |
| Sausage | HvL | Hongcheng Zhu,2013 | 3/6/9 | 179,773 | 3,293 | RR | 1.33 (1.16, 1.52) | 0.002^*^ | 59 | 0.012 | 0.028 |
| White meat | HvL | Seong Rae Kim,2019 | 5/16/21 | 1,632,077 | 9,896 | RR | 0.80 (0.69, 0.92) | 0.002^*^ | 41.9 | 0.023 | 0.116 |
| White meat | 100 g/d increment | Seong Rae Kim,2019 | 4/10/14 | 1,587,168 | 6,802 | RR | 0.86 (0.64, 1.15) | 0.303^*^ | 52.8 | 0.01 | 0.096 |
| Fish | HvL | Shengjun Wu,2011 | 2/15/17 | 136,226 | 5,323 | RR | 0.87 (0.71，1.07) | 0.174^*^ | 73.3 | <0.001 | 0.59 |
| Salt | HvL | Sheng Ge,2012 | 7/4/11 | 2076498 | 12,039 | OR | 2.05 (1.60, 2.62) | <0.001 | 92 | <0.001 | 0.005 |
| Pickled foods | HvL | Lanfranco D'Elia,2012 | 11/0/11 | 242,568 | 2,858 | RR | 1.27 (1.09, 1.49) | 0.002 | 25 | 0.2 | 0.039^*^ |
| Salted fish | HvL | Lanfranco D'Elia,2012 | 13/0/13 | 209,704 | 1,447 | RR | 1.24 (1.03, 1.50) | 0.022 | 0 | 0.75 | 0.656^*^ |
| Miso-soup | HvL | Lanfranco D'Elia,2012 | 12/0/12 | 249,931 | 3,022 | RR | 1.05 (0.88, 1.25) | 0.59 | 27 | 0.18 | 0.821^*^ |
| Nitrite intake | HvL | Fei-Xiong Zhang,2019 | 5/14/19 | 787,050 | 11,901 | OR | 1.27 (1.03, 1.55) | 0.022 | 89.6 | <0.001 | 0.061 |
| Nitrite intake | MvL | Fei-Xiong Zhang,2019 | 5/10/15 | 781,427 | 9,962 | OR | 1.12 (1.01, 1.26) | 0.037 | 63.9 | <0.001 | 0.115 |
| Nitrate intake | HvL | Fei-Xiong Zhang,2019 | 5/12/17 | 768,937 | 9,028 | OR | 0.81 (0.68, 0.97) | 0.021 | 76.3 | <0.001 | 0.054 |
| Nitrate intake | MvL | Fei-Xiong Zhang,2019 | 5/10/15 | 768,211 | 8,704 | OR | 0.86 (0.75, 0.99) | 0.036 | 67.6 | <0.001 | 0.323 |
| Nitrosamines | HvL | Peng Song,2015 | 7/4/11 | NA | NA | RR | 1.34 (1.02, 1.76) | 0.032^*^ | 75.8^*^ | <0.001^*^ | 0.692^*^ |
| Vegetable | HvL | Qingbing Wang,2014 | 19/0/19 | 2,408,505 | 6,005 | RR | 0.96 (0.88, 1.06) | 0.413^*^ | 21.1 | 0.198 | 0.152 |
| Vegetable | 100 g/d increment | Qingbing Wang,2014 | 16/0/16 | 1,420,827 | 4,581 | RR | 0.96 (0.91, 1.01) | 0.135^*^ | 49.7 | 0.012 | 0.057^*^ |
| Fruit | HvL | Qingbing Wang,2014 | 22/0/22 | 1,517,969 | 5,318 | RR | 0.90 (0.83, 0.98) | 0.016^*^ | 0.7 | 0.45 | 0.191 |
| Fruit | 100 g/d increment | Qingbing Wang,2014 | 16/0/16 | 1,465,757 | 4,899 | RR | 0.95 (0.91, 0.99) | 0.01^*^ | 38 | 0.062 | 0.373^*^ |
| Citrus fruit | HvL | Paola Bertuccio,2019 | 0/15/15 | 20,830 | 6,340 | OR | 0.80 (0.73, 0.87) | 0.023^*^ | 74.7^*^ | <0.001^*^ | 0.411 |
| Allium vegetable | HvL | Yong Zhou,2011 | 2/19/21 | 543,220 | 7,644 | OR | 0.54 (0.43, 0.65) | <0.001 | 83.6 | 0.001 | 0.11 |
| Garlic | HvL | Federica Turati,2015 | 0/12/12 | NA | 3,807 | RR | 0.60 (0.47, 0.76) | <0.001^*^ | 54.9 | 0.01 | 0.86^*^ |
| Onion | HvL | Federica Turati,2015 | 0/13/13 | NA | 4,619 | RR | 0.55 (0.41, 0.73) | <0.001^*^ | 76 | <0.001 | 0.04 |
| Cruciferous vegetable | HvL | Qi-Jun Wu,2013 | 6/16/22 | 1,406,973 | 7,594 | RR | 0.81 (0.75, 0.88) | <0.001^*^ | 12 | 0.292 | 0.668 |
| Cabbage | HvL | Qi-Jun Wu,2013 | 2/5/7 | 643,198 | 2,289 | RR | 0.68 (0.58, 0.80) | <0.001^*^ | 0 | 0.449 | 0.125 |
| Carrot | HvL | Hossein Fallahzadeh,2015 | 2/3/5 | 227,173 | 3,867 | OR | 0.74 (0.68, 0.81) | <0.001 | 0 | 0.576 | 0.016 |
| Tomato | HvL | Tingsong Yang, 2013 | 0/7/7 | 10,089 | 2,596 | OR | 0.73 (0.60, 0.90) | 0.001^*^ | 47.92 | 0.07 | 0.44 |
| Nuts | HvL | Dai Zhang,2020 | 2/3/5 | 735,356 | 10,468 | RR | 0.83 (0.71, 0.97) | 0.017^*^ | 54.4 | 0.067 | 0.642^*^ |
| Total soy products | HvL | Yameng Wang,2021 | 2/11/13 | 125,219 | 5,354 | RR | 0.64 (0.51, 0.80) | <0.001^*^ | 77.4 | <0.001 | 0.05 |
| Non-fermented soybean products | HvL | Yameng Wang,2021 | 5/6/11 | 255,917 | 4,481 | RR | 0.79 (0.71, 0.87) | <0.001^*^ | 50.6 | 0.004 | <0.001 |
| Fermented soybean products | HvL | Yameng Wang,2021 | 8/7/15 | 307,647 | 7,258 | RR | 1.19 (1.02, 1.38) | 0.023^*^ | 65.8 | <0.001 | 0.17 |
| Chili | M-HvL | Yanbin Du,2020 | 0/13/13 | 7856 | 3,095 | OR | 1.96 (1.59, 2.42) | <0.001^*^ | 74.7 | <0.001 | 0.288 |
| Total alcohol | HvL | Peng-Liang Wang,2017 | 17/56/73 | 2,080,531 | 36,499 | RR | 1.25 (1.15, 1.37) | <0.001 | 68.8 | <0.001 | 0.261 |
| Total alcohol | 12.5 g/d increment | Peng-Liang Wang,2017 | 13/15/28 | 1,753,950 | 13,709 | RR | 1.04 (1.01, 1.07) | 0.005 | 67.5 | <0.001 | 0.106^*^ |
| Beer | HvL | Peng-Liang Wang,2017 | 7/17/24 | 1,071,478 | 7,901 | RR | 1.13 (1.03, 1.24) | 0.012 | 9.4 | 0.331 | 0.098^*^ |
| Beer | 12.5 g/d increment | Peng-Liang Wang,2017 | 5/9/14 | 996,281 | 5,547 | RR | 1.07 (1.01, 1.13) | 0.025 | 9.5 | 0.348 | 0.81^*^ |
| Liquor | HvL | Peng-Liang Wang,2017 | 8/20/28 | 1,099,994 | 9,703 | RR | 1.22 (1.06, 1.40) | 0.005 | 53.6 | <0.001 | 0.448^*^ |
| Liquor | 12.5 g/d increment | Peng-Liang Wang,2017 | 5/9/14 | 996,617 | 5,852 | RR | 1.03 (0.98, 1.09) | 0.296 | 24.1 | 0.193 | 0.86^*^ |
| Wine | HvL | Peng-Liang Wang,2017 | 8/18/26 | 1,102,795 | 9,450 | RR | 0.99 (0.84, 1.16) | 0.857 | 54.6 | <0.001 | 0.988^*^ |
| Wine | 12.5 g/d increment | Peng-Liang Wang,2017 | 4/9/13 | 988,141 | 5,397 | RR | 0.99 (0.93, 1.06) | 0.769 | 61.9 | 0.002 | 0.011^*^ |
| Tea | HvL | Long-Gang Zhao,2021 | 4/0/4 | NA | 23,764 | RR | 1.02 (0.81, 1.27) | 0.876 | 53.9 | 0.089 | 0.106 |
| Tea | 1 cup/d increment | Long-Gang Zhao,2021 | 4/0/4 | NA | 23,734 | RR | 0.98 (0.94, 1.03) | 0.456 | 22.5 | 0.276 | 0.332 |
| Black tea | HvL | Long-Gang Zhao,2021 | 4/0/4 | NA | 1,167 | RR | 1.08 (0.83, 1.42) | 0.549 | 15.8 | 0.313 | 0.115 |
| Black tea | 1 cup/d increment | Long-Gang Zhao,2021 | 3/0/3 | NA | 1,059 | RR | 1.01 (0.85, 1.19) | 0.939 | 5.4 | 0.347 | 0.35 |
| Green tea | HvL | Yanhong Huang,2017 | 5/8/13 | 376,943 | 6,627 | RR | 0.89(0.76, 1.03)^*^ | 0.119^*^ | 49.2^*^ | 0.023^*^ | 0.124^*^ |
| Green tea | 1 cup/d increment | Long-Gang Zhao,2021 | 4/0/4 | NA | 4,965 | RR | 1.00 (0.96, 1.03) | 0.83 | 62.8 | 0.045 | 0.166 |
| Coffee | HvL | Feiyue Xie,2014 | 12/0/12 | 840,651 | 2,688 | RR | 1.12 (0.93, 1.36) | 0.244 | 37 | 0.074 | 0.037 |
| Sugar-sweetened beverages | HvL | Yuting Li,2021 | 2/4/6 | 523,488 | 2,334 | RR | 0.99 (0.79, 1.29) | 0.96 | 48.3 | 0.043 | 0.062^*^ |
| Dairy product | HvL | Yan Sun,2014 | 10/27/37 | 110,046 | 8,514 | RR | 1.06 (0.95, 1.18) | 0.295^*^ | 67.1 | 0 | 0.135 |
| Milk | HvL | Yan Sun,2014 | 7/16/23 | NA | NA | RR | 1.11 (0.94, 1.31) | 0.206^*^ | 70.1 | 0 | 0.183^*^ |
| Cheese | HvL | Yan Sun,2014 | 2/7/9 | NA | NA | RR | 0.95 (0.80, 1.12) | 0.591^*^ | 9.1 | 0.36 | 0.621 |
| **Micronutrients** |  |  |  |  |  |  |  |  |  |  |  |
| Total Vitamin | HvL | Pengfei Kong,2014 | 7/29/36 | 1,068,544 | 11,207 | RR | 0.73(0.68, 0.78)^*^ | <0.001^*^ | 47.6^*^ | 0.001^*^ | 0.304^*^ |
| Retinol | HvL | YihuaWu,2015 | 4/15/19 | 253,776 | 5,706 | RR | 0.94 (0.87, 1.03) | 0.116^*^ | 45.3 | 0.017 | 0.123 |
| Vitamin A | HvL | YihuaWu,2015 | 2/13/15 | 96,023 | 2,877 | RR | 0.66 (0.52, 0.84) | 0.001^*^ | 64.6 | <0.001 | 0.35 |
| Vitamin A | 1.5 mg/d increment | Pengfei Kong,2014 | 2/6/8 | 128,166 | 1,772 | RR | 0.71 (0.62, 0.81) | <0.001^*^ | 22 | 0.25 | 0.664^*^ |
| Vitamin D | HvL | Saeid Khayatzadeh,2015 | 0/4/4 | 5,725 | 1,652 | OR | 1.09 (0.94, 1.25) | 0.26 | 13.2 | 0.33 | 0.35 |
| Vitamin C | HvL | Peiwei Li,2014 | 4/28/32 | 733,894 | 9,455 | OR | 0.58 (0.51, 0.65) | <0.001^*^ | 46.7 | 0.002 | 0.675^*^ |
| Vitamin C | 100 mg/d increment | Pengfei Kong,2014 | 2/9/11 | 268,253 | 4,871 | RR | 0.74 (0.69, 0.79) | <0.001^*^ | 4 | 0.4 | 0.707^*^ |
| Vitamin E | HvL | Peiwei Li,2014 | 4/20/24 | 694,806 | 7,095 | OR | 0.65 (0.57, 0.74) | <0.001^*^ | 48.3 | 0.005 | 0.063^*^ |
| Vitamin E | 10 mg/d increment | Pengfei Kong,2014 | 3/5/8 | 755,519 | 3,581 | RR | 0.76 (0.67, 0.85) | <0.001^*^ | 43 | 0.09 | 0.557^*^ |
| β-carotene | HvL | Peiwei Li,2014 | 3/17/20 | 245,858 | 6,258 | OR | 0.59 (0.49, 0.70) | <0.001^*^ | 68.7 | <0.001 | 0.827^*^ |
| α-carotene | HvL | Peiwei Li,2014 | 2/6/8 | 206,345 | 1,614 | OR | 0.69 (0.52, 0.93) | 0.014^*^ | 58.4 | 0.019 | 0.787^*^ |
| Folate | HvL | Martin Tio,2014 | 3/13/16 | 20,968 | 4,414 | OR | 0.94 (0.78, 1.14) | 0.83 | 55.1 | 0.003 | 0.31 |
| Selenium | HvL | Marco Vinceti,2018 | 5/0/5 | 197,000 | 955 | OR | 0.66 (0.43, 1.01) | 0.053^*^ | 51 | 0.06 | 0.988^*^ |
| Zinc | HvL | Peiwei Li,2014 | 0/7/7 | 4,004 | 1,150 | RR | 0.91 (0.64, 1.29) | 0.581 | 77.6 | <0.001 | 0.575^*^ |
| Total Polyphenols | HvL | Facundo Vitelli-Storelli,2020 | 0/10/10 | 11,815 | 3,471 | OR | 0.67 (0.54, 0.81) | 0.001^*^ | 62.3 | 0.004 | 0.721^*^ |
| Isoflavones | HvL | Jie You,2018 | 6/6/12 | 596,553 | 3,190 | OR | 0.97 (0.87, 1.09) | 0.72^*^ | 27.5 | 0.174 | 0.957 |
| Flavonoid | HvL | Yacong Bo,2016 | 3/3/6 | 516,308 | 2,145 | OR | 0.88 (0.74, 1.04) | 0.212^*^ | 63.6 | 0.007 | 0.17 |
| Anthocyanins | HvL | DeYi Yang，2020 | 2/4/6 | 951,206 | 3,243 | RR | 0.92 (0.81, 1.04) | 0.177^*^ | 0 | 0.597 | 0.425 |
| **Use of medication therapy** | |  |  |  |  |  |  |  |  |  |  |
| PPI | regular vs. not | Ruijie Zeng,2021 | 6/5/11 | 3,015,141 | 48,589 | RR | 1.78 (1.38, 2.31) | <0.001^*^ | 95.2 | <0.001 | 0.635^*^ |
| Aspirin | regular vs. not | C Bosetti，2020 | 6/8/14 | NA | 9,919 | RR | 0.64 (0.51, 0.82) | <0.001 | 91 | <0.001 | 0.268 |
| Aspirin | ≥5 yrs vs. not | Lijuan Wang,2021 | 3/0/3 | 890,956 | 6,164 | RR | 0.60 (0.38, 0.94) | 0.027 | 86 | 0.001^*^ | 0.102^*^ |
| Metformin | ever vs.never | Y.Shuai,2020 | 8/0/8 | 1,238,382 | NA | HR | 0.79 (0.62, 1.00) | 0.051 | 88.3 | <0.001 | 0.635^*^ |
| Bisphosphonates | ever vs.never | Ellen Wright,2015 | 4/0/4 | 105,215 | 7,133 | OR | 0.96 (0.82, 1.12) | 0.774^*^ | 30.3 | 0.231 | 0.654^*^ |
| Statins | ever vs.never | P P Singh,2013 | 1/7/8 | 5,311,451 | 5,290 | OR | 0.65 (0.45, 0.93) | 0.018^*^ | 92.4^*^ | <0.001^*^ | 0.449^*^ |
| Menopausal hormone therapy | ever vs.never | M Constanza Camargo,2012 | 4/3/7 | NA | 2,152 | RR | 0.77 (0.64, 0.92) | 0.005^*^ | 0 | 0.99 | 0.95 |
| **Lifestyle** |  |  |  |  |  |  |  |  |  |  |  |
| Smoking | ever vs. never | Ana Ferro,2018 | 23/0/23 | 36,435 | 10,290 | OR | 1.20 (1.09, 1.32) | <0.001^*^ | 48.2 | 0.005^*^ | 0.796^*^ |
| Smoking | current vs.never | Ricardo Ladeiras-Lopes,2008 | 23/0/23 | NA | NA | RR | 1.53 (1.42, 1.65) | <0.001^*^ | 57.5 | <0.001^*^ | 0.513^*^ |
| Smoking | former vs.never | Ricardo Ladeiras-Lopes,2008 | 22/0/22 | NA | NA | RR | 1.30 (1.21, 1.40) | <0.001^*^ | 28 | 0.079^*^ | 0.877^*^ |
| Sedentary Behavior | HvL | Daniela Schmid,2014 | 3/2/5 | 721,505 | 2,672 | RR | 1.05 (0.87, 1.26) | 0.649^*^ | 0 | 0.76 | 0.635^*^ |
| Physical activity | HvL | Theodora Psaltopoulou,2016 | 10/12/22 | 1,620,640 | 13,354 | RR | 0.81 (0.73, 0.89) | <0.001^*^ | 53.1 | 0.001 | 0.24 |
| Toothbrushing frequency | HvL | Huadong Wu,2021 | 2/1/3 | 218,120 | 4,434 | OR | 0.84 (0.77, 0.92) | 0.015^*^ | 30.8 | 0.236 | 0.4^*^ |
| Refrigerator use | yes vs.no | Shijiao Yan,2018 | 1/11/12 | 14,361 | 3987 | OR | 0.70 (0.56, 0.88) | <0.001 | 89.8 | <0.001 | 0.183 |
| **Pre-existing medical conditions** | |  |  |  |  |  |  |  |  |  |  |
| Depression | present vs. absent | Yuehua Zhang,2021 | 2/22/24 | 104,074 | 48,592 | OR | 1.84 (1.61, 2.09) | <0.001 | 75 | <0.001 | 0.177 |
| NAFLD | present vs. absent | Shou-Sheng Liu,2020 | 3/0/3 | 73,054 | 240 | OR | 1.74 (1.03, 2.95) | 0.011^*^ | 73.6 | 0.01 | 0.057^*^ |
| SLE | present vs. absent | Ann EClarkeMD,2021 | 9/0/9 | 72,444 | 51 | RR | 1.34 (1.05, 1.72) | 0.02^*^ | 0 | 0.8 | 0.12^*^ |
| IBD | present vs. absent | Qianyi Wan,2021 | 6/1/7 | 319,235 | 108 | OR | 0.87 (0.62, 1.13) | 0.820^*^ | 0 | 0.605^*^ | 0.895^*^ |
| Pernicious anemia | present vs. absent | Minkyo Song,2019 | 12/2/14 | NA | 2,688 | RR | 2.84 (2.30, 3.50) | <0.001^*^ | 82.4 | <0.001 | 0.32 |
| Diabetes mellitus,type 1 | present vs. absent | Minkyo Song,2019 | 8/1/9 | NA | 256 | RR | 1.41 (1.20, 1.67) | <0.001^*^ | 35.7 | 0.13 | 0.89 |
| Diabetes mellitus | present vs. absent | T Tian,2012 | 14/7/21 | NA | 7,715 | RR | 1.11 (1.00, 1.24) | 0.045 | 79.5 | <0.001 | 0.858^*^ |
| GDM | present vs. absent | Y Wang,2020 | 3/0/3 | 1,278,787 | 607 | RR | 1.43 (1.02, 2.00) | 0.039 | 0 | 0.537 | 0.064^*^ |
| **Viral or bacterial infection** | |  |  |  |  |  |  |  |  |  |  |
| HP | present vs. absent | Yoon Park,2021 | 0/11/11 | 13,010 | 7,099 | OR | 1.69 (1.29, 2.22) | <0.001^*^ | 84.2 | <0.001 | 0.857^*^ |
| EBV | present vs. absent | Jong-Myon Bae,2016 | 0/14/14 | 1,393 | 945 | OR | 3.41 (1.78, 6.51) | <0.001^*^ | 65.5 | 0.001^*^ | 0.183^*^ |
| HBV | present vs. absent | Yusha Yang,2021 | 5/5/10 | NA | NA | HR | 1.26 (1.08, 1.47) | 0.003 | 89.3 | <0.001 | 0.455 |
| HCV | present vs. absent | Yusha Yang,2021 | 5/0/5 | NA | NA | HR | 1.88 (1.28, 2.76) | 0.001 | 74.7 | 0.003 | 0.228 |
| HCMV | present vs. absent | Hui Wang,2020 | 0/0/5^#^ | 682 | 265 | OR | 2.09 (1.14, 3.84) | 0.016^*^ | 40.4^*^ | 0.058 | 0.071^*^ |
| HPV | present vs. absent | Hui Wang,2020 | 0/0/14^#^ | 2,106 | 901 | OR | 1.53 (1.00, 2.33) | 0.047^*^ | 59.0^*^ | 0.003^*^ | 0.355^*^ |
| HTLV-1 | present vs. absent | Hui Wang,2020 | 0/0/2^#^ | 4,293 | 463 | OR | 0.89 (0.61, 1.30) | 0.611^*^ | 17^*^ | 0.272 | NA |
| JCV | present vs. absent | Hui Wang,2020 | 0/0/9^#^ | 1,356 | 692 | OR | 2.28 (1.14, 4.56) | 0.009^*^ | 51.4^*^ | 0.011^*^ | 0.246^*^ |
| **Other factors** |  |  |  |  |  |  |  |  |  |  |  |
| Education level | HvL | Matteo Rota,2020 | 0/24/24 | 5,533 | 1,024 | OR | 0.60 (0.44, 0.84) | 0.004^*^ | 85.5 | <0.001^*^ | 0.781^*^ |
| Household income | HvL | Matteo Rota,2020 | 0/6/6 | 1,582 | 323 | OR | 0.65 (0.48, 0.89) | 0.006^*^ | 52^*^ | 0.064^*^ | 0.681^*^ |
| ABO blood group | blood group A vs non-A | Zhiwei Wang,2012 | 1/23/24 | 1,421,740 | 15,843 | OR | 1.11 (1.07, 1.15) | <0.001^*^ | 62.4 | <0.001 | 0.77 |
| ABO blood group | blood group 0 vs non-0 | Zhiwei Wang,2012 | 1/23/24 | 1,421,740 | 15,843 | OR | 0.91 (0.89, 0.94) | <0.001^*^ | 34.6^*^ | 0.044^*^ | 0.27 |
| Tooth Loss | HvL | Xin-Hai Yin,2016 | 5/4/9 | 153,093 | 3,113 | RR | 1.44 (1.05, 1.98) | 0.001 | 71.3 | 0.001 | 0.9 |
| PM2.5 | Per 5 μg/m3 increase | Gabriele Nagel,2018 | 8/0/8 | 219,397 | 660 | HR | 1.38 (0.99, 1.92) | 0.057^*^ | 0 | 0.97 | 0.094^*^ |
| Years of fertility | HvL | M Constanza Camargo,2012 | 4/4/8 | 497,326 | 2,118 | RR | 0.74 (0.63, 0.86) | <0.001^*^ | 0 | 0.76 | 0.29 |

**Abbreviations:** BMI, body mass index; DII, dietary inflammatory index; D-TAC, dietary total antioxidant capacity; PPI, proton pump inhibitors; NAFLD, non-alcoholic fatty liver disease; SLE, systemic lupus erythematosus; IBD, inflammatory bowel disease; GDM, gestational diabetes mellitus; HP, Helicobacter pylori; EBV, Epstein-Barr virus; HBV, hepatitis B virus; HCV, hepatitis C virus; HCMV, human cytomegalovirus; HPV, human papillomavirus; HTLV-1, human T-cell lymphotropic virus type 1; JCV, John Cunningham virus; PM2.5: particulate matter with a diameter of 2.5 μm or less; HvL, highest versus lowest; NA, not applicable.

^#^ including cross-sectional studies *The result was reanalyzed
