# Supplementary material for: Risk factors for gastric cancer: A comprehensive analysis of observational studies
Source: Front Public Health. 2023 Jan 4;10:892468. doi: 10.3389/fpubh.2022.892468 (PMC9845896; doi:10.3389/fpubh.2022.892468)
Supplement: Supplementary file 2 [file Table_2.DOCX]

**Supplementary Table2. Assessments of AMSTAR 2 scores.**

| **Author, year** | **AMSTAR2 checklist** | | | | | | | | | | | | | | | | **Overall assessment quality** |
| --- | --- | --- | --- | --- | --- | --- | --- | --- | --- | --- | --- | --- | --- | --- | --- | --- | --- |
|  | **NO.1** | **NO.2** | **NO.3** | **NO.4** | **NO.5** | **NO.6** | **NO.7** | **NO.8** | **NO.9** | **NO.10** | **NO.11** | **NO.12** | **NO.13** | **NO.14** | **NO.15** | **NO.16** |  |
| Min Seok Seo,2020 | Yes | No | Yes | Partial yes | Yes | Yes | Partial yes | Yes | Yes | No | Yes | Yes | Yes | Yes | Yes | Yes | Critically low |
| Xue-Jun Lin,2014 | Yes | No | Yes | Partial yes | Yes | Yes | Partial yes | Partial yes | Yes | No | Yes | Yes | Yes | Yes | Yes | Yes | Critically low |
| Xuan Du,2017 | Yes | No | Yes | Partial yes | No | No | Partial yes | Yes | Yes | No | Yes | Yes | Yes | No | Yes | Yes | Critically low |
| Jakub Morze,2021 | Yes | Yes | Yes | Partial yes | Yes | Yes | Partial yes | Yes | Yes | No | Yes | No | No | No | Yes | Yes | Critically low |
| Ying Liang,2019 | Yes | No | Yes | Partial yes | Yes | Yes | Yes | Yes | Yes | No | Yes | Yes | Yes | Yes | Yes | Yes | Critically low |
| Federica Turati,2019 | Yes | No | Yes | Partial yes | Yes | Yes | Partial yes | Yes | No | No | Yes | No | No | Yes | Yes | Yes | Critically low |
| MohammadParohan,2019 | Yes | No | Yes | Partial yes | Yes | Yes | Partial yes | Yes | Yes | No | Yes | Yes | Yes | Yes | Yes | Yes | Critically low |
| Yao Ye,2017 | Yes | No | Yes | Partial yes | Yes | Yes | Partial yes | Partial yes | Yes | No | Yes | No | No | Yes | Yes | Yes | Critically low |
| Zhizhong Zhang,2013 | Yes | No | Yes | Partial yes | Yes | Yes | Partial yes | Partial yes | Yes | No | Yes | Yes | Yes | Yes | Yes | Yes | Critically low |
| Xiao-Feng Zhang,2020 | Yes | No | Yes | Partial yes | Yes | Yes | Partial yes | Partial yes | Yes | No | Yes | Yes | Yes | Yes | Yes | Yes | Critically low |
| Tonghua Wang,2020 | Yes | No | Yes | Partial yes | Yes | Yes | Partial yes | Partial yes | Yes | No | Yes | Yes | No | Yes | Yes | Yes | Critically low |
| Peng Miao,2021 | Yes | Yes | Yes | Partial yes | Yes | Yes | Partial yes | Partial yes | Yes | No | Yes | Yes | No | Yes | Yes | Yes | Critically low |
| Jun Han,2015 | Yes | No | Yes | Partial yes | Yes | Yes | Partial yes | Partial yes | Yes | No | Yes | Yes | No | Yes | Yes | Yes | Critically low |
| Ana Ferro,2019 | Yes | No | Yes | Partial yes | No | No | Partial yes | Partial yes | No | No | Yes | No | No | Yes | No | Yes | Critically low |
| Seong Rae Kim,2019 | Yes | No | Yes | Partial yes | Yes | Yes | Partial yes | Yes | Yes | No | Yes | Yes | Yes | Yes | No | Yes | Critically low |
| Hongcheng Zhu,2013 | Yes | No | Yes | Partial yes | Yes | Yes | Partial yes | Yes | Yes | No | Yes | No | No | Yes | Yes | Yes | Critically low |
| Shengjun Wu,2011 | Yes | No | Yes | No | Yes | Yes | Partial yes | Yes | No | No | Yes | No | Yes | Yes | No | Yes | Critically low |
| Sheng Ge,2012 | Yes | No | Yes | Partial yes | Yes | Yes | Yes | Yes | Yes | No | Yes | Yes | No | Yes | No | Yes | Critically low |
| Lanfranco D'Elia,2012 | Yes | No | Yes | Partial yes | Yes | Yes | Yes | Yes | Yes | No | Yes | Yes | Yes | Yes | No | Yes | Critically low |
| Fei-Xiong Zhang,2019 | Yes | No | Yes | Partial yes | Yes | Yes | Partial yes | Partial yes | Yes | No | Yes | Yes | Yes | Yes | Yes | Yes | Critically low |
| Peng Song,2015 | Yes | No | Yes | Partial yes | Yes | Yes | Partial yes | Yes | Yes | No | Yes | Yes | Yes | Yes | Yes | Yes | Critically low |
| Qingbing Wang,2014 | Yes | No | Yes | Partial yes | Yes | Yes | Partial yes | Yes | Yes | No | Yes | Yes | Yes | Yes | Yes | Yes | Critically low |
| Paola Bertuccio,2019 | Yes | No | Yes | Partial yes | Yes | Yes | Partial yes | Partial yes | Yes | No | Yes | Yes | No | Yes | No | Yes | Critically low |
| Yong Zhou,2011 | Yes | No | Yes | Partial yes | Yes | Yes | Yes | Partial yes | No | No | Yes | No | No | Yes | Yes | Yes | Critically low |
| Federica Turati,2015 | Yes | No | Yes | Partial yes | Yes | Yes | Yes | Partial yes | No | No | Yes | No | No | Yes | Yes | Yes | Critically low |
| Qi-Jun Wu,2013 | Yes | No | Yes | Partial yes | Yes | Yes | Partial yes | Yes | Yes | No | Yes | Yes | Yes | Yes | Yes | Yes | Critically low |
| Hossein Fallahzadeh,2015 | Yes | No | Yes | Partial yes | No | No | Partial yes | Partial yes | No | No | Yes | No | No | Yes | Yes | Yes | Critically low |
| Tingsong Yang, 2013 | Yes | No | Yes | Partial yes | Yes | Yes | Partial yes | Partial yes | No | No | Yes | No | No | Yes | Yes | No | Critically low |
| Dai Zhang,2020 | Yes | No | Yes | Partial yes | Yes | Yes | Partial yes | Yes | Yes | No | Yes | Yes | Yes | Yes | Yes | Yes | Critically low |
| Yameng Wang,2021 | Yes | Yes | Yes | Partial yes | No | Yes | Partial yes | Yes | Yes | No | Yes | Yes | No | Yes | No | Yes | Critically low |
| Yanbin Du,2020 | Yes | Yes | Yes | Partial yes | Yes | Yes | Partial yes | Yes | Yes | No | Yes | Yes | Yes | Yes | Yes | Yes | Critically low |
| Peng-Liang Wang,2017 | Yes | No | Yes | No | Yes | Yes | Partial yes | Yes | Yes | No | Yes | Yes | Yes | Yes | No | Yes | Critically low |
| Long-Gang Zhao,2021 | Yes | Yes | Yes | Partial yes | Yes | Yes | Yes | Yes | Yes | No | Yes | Yes | Yes | Yes | Yes | Yes | Low |
| Yanhong Huang,2017 | Yes | No | Yes | Partial yes | Yes | Yes | Partial yes | Partial yes | Yes | No | Yes | Yes | Yes | Yes | Yes | Yes | Critically low |
| Feiyue Xie,2014 | Yes | No | Yes | Partial yes | Yes | Yes | Partial yes | Partial yes | No | No | Yes | Yes | Yes | Yes | Yes | Yes | Critically low |
| Yuting Li,2021 | Yes | No | Yes | Partial yes | Yes | Yes | Partial yes | Yes | Yes | No | Yes | Yes | Yes | Yes | Yes | Yes | Critically low |
| Yan Sun,2014 | Yes | No | Yes | Partial yes | No | No | Partial yes | Yes | Yes | No | Yes | Yes | Yes | Yes | Yes | Yes | Critically low |
| Pengfei Kong,2014 | Yes | No | Yes | Partial yes | Yes | Yes | Partial yes | Partial yes | Yes | No | Yes | Yes | No | Yes | No | Yes | Critically low |
| YihuaWu,2015 | Yes | No | Yes | Partial yes | Yes | Yes | Partial yes | Yes | Yes | No | Yes | No | No | Yes | Yes | Yes | Critically low |
| Saeid Khayatzadeh,2015 | Yes | No | Yes | Partial yes | Yes | Yes | Partial yes | Yes | No | No | Yes | No | No | Yes | No | Yes | Critically low |
| Peiwei Li,2014 | Yes | No | Yes | Partial yes | Yes | Yes | Partial yes | Partial yes | Yes | No | Yes | No | No | Yes | Yes | Yes | Critically low |
| Martin Tio,2014 | Yes | No | Yes | Partial yes | Yes | Yes | Partial yes | Partial yes | No | No | Yes | Yes | Yes | Yes | Yes | Yes | Critically low |
| Marco Vinceti,2018 | Yes | Yes | Yes | Yes | Yes | Yes | Partial yes | Yes | Yes | No | Yes | Yes | Yes | Yes | Yes | Yes | Low |
| Peiwei Li,2014 | Yes | No | Yes | Partial yes | Yes | Yes | Partial yes | Partial yes | Yes | No | Yes | Yes | Yes | Yes | Yes | Yes | Critically low |
| Facundo Vitelli-Storelli,2020 | Yes | No | Yes | Partial yes | Yes | Yes | Partial yes | Partial yes | No | No | Yes | No | No | No | No | Yes | Critically low |
| Jie You,2018 | Yes | No | Yes | Partial yes | Yes | Yes | Partial yes | Partial yes | Yes | No | Yes | No | No | Yes | Yes | Yes | Critically low |
| Yacong Bo,2016 | Yes | No | Yes | Partial yes | Yes | Yes | Partial yes | Partial yes | No | No | Yes | Yes | Yes | Yes | Yes | Yes | Critically low |
| DeYi Yang，2020 | Yes | No | Yes | Partial yes | Yes | Yes | Partial yes | Yes | Yes | No | Yes | Yes | No | Yes | No | Yes | Critically low |
| Ruijie Zeng,2021 | Yes | Yes | Yes | Partial yes | Yes | Yes | Partial yes | Yes | Yes | No | Yes | No | No | Yes | Yes | Yes | Critically low |
| C Bosetti，2020 | Yes | Yes | Yes | Partial yes | Yes | Yes | Yes | Yes | No | No | Yes | Yes | Yes | Yes | Yes | Yes | Critically low |
| Lijuan Wang,2021 | Yes | No | Yes | Partial yes | Yes | Yes | Partial yes | Partial yes | No | No | Yes | No | No | Yes | Yes | Yes | Critically low |
| Y.Shuai,2020 | Yes | No | Yes | Partial yes | Yes | Yes | Yes | Yes | Yes | No | Yes | No | No | Yes | Yes | Yes | Critically low |
| Ellen Wright,2015 | Yes | No | Yes | Partial yes | No | No | Partial yes | Yes | No | No | Yes | Yes | Yes | Yes | No | Yes | Critically low |
| P P Singh,2013 | Yes | No | Yes | Partial yes | Yes | Yes | Partial yes | Yes | Yes | No | Yes | Yes | Yes | Yes | Yes | Yes | Critically low |
| M Constanza Camargo,2012 | Yes | No | Yes | Partial yes | Yes | Yes | Yes | Yes | No | No | Yes | Yes | No | Yes | Yes | Yes | Critically low |
| Ana Ferro,2018 | Yes | No | Yes | Partial yes | Yes | Yes | Partial yes | Yes | No | No | Yes | No | No | Yes | Yes | Yes | Critically low |
| Ricardo Ladeiras-Lopes,2008 | Yes | No | Yes | Partial yes | Yes | Yes | Yes | Partial yes | No | No | Yes | Yes | Yes | Yes | Yes | Yes | Critically low |
| Daniela Schmid,2014 | Yes | No | Yes | Partial yes | Yes | Yes | Partial yes | Partial yes | Yes | No | Yes | No | Yes | Yes | No | Yes | Critically low |
| Theodora Psaltopoulou,2016 | Yes | No | Yes | Partial yes | Yes | Yes | Partial yes | Yes | Yes | No | Yes | No | No | Yes | Yes | Yes | Critically low |
| Huadong Wu,2021 | Yes | No | Yes | Partial yes | Yes | Yes | Partial yes | Partial yes | Yes | No | Yes | Yes | Yes | Yes | No | Yes | Critically low |
| Shijiao Yan,2018 | Yes | No | Yes | Partial yes | Yes | Yes | Partial yes | Yes | Yes | No | Yes | Yes | Yes | Yes | Yes | Yes | Critically low |
| Yuehua Zhang,2021 | Yes | No | Yes | Partial yes | Yes | Yes | Partial yes | Partial yes | Yes | No | Yes | Yes | Yes | Yes | Yes | Yes | Critically low |
| Shou-Sheng Liu,2020 | Yes | No | Yes | Partial yes | Yes | Yes | Partial yes | Partial yes | Yes | No | Yes | No | No | Yes | No | Yes | Critically low |
| Ann EClarkeMD,2021 | Yes | Yes | Yes | Partial yes | Yes | Yes | Yes | Yes | Yes | No | Yes | Yes | Yes | Yes | Yes | Yes | Low |
| Qianyi Wan,2021 | Yes | Yes | Yes | Partial yes | Yes | Yes | Partial yes | Partial yes | Yes | No | Yes | Yes | Yes | Yes | Yes | Yes | Critically low |
| Minkyo Song,2019 | Yes | No | Yes | Partial yes | Yes | Yes | Partial yes | Partial yes | Yes | No | Yes | Yes | No | Yes | Yes | Yes | Critically low |
| T Tian,2012 | Yes | No | Yes | Partial yes | Yes | Yes | Partial yes | Partial yes | No | No | Yes | No | No | Yes | Yes | Yes | Critically low |
| Y Wang,2020 | Yes | No | Yes | Partial yes | Yes | Yes | Partial yes | Yes | Yes | No | Yes | No | No | Yes | Yes | Yes | Critically low |
| Yoon Park,2021 | Yes | No | Yes | Partial yes | Yes | Yes | Partial yes | Partial yes | No | No | Yes | No | No | Yes | No | Yes | Critically low |
| Jong-Myon Bae,2016 | Yes | Yes | Yes | Partial yes | No | Yes | Partial yes | Partial yes | Yes | No | Yes | No | No | Yes | Yes | Yes | Critically low |
| Yusha Yang,2021 | Yes | Yes | Yes | Partial yes | Yes | Yes | Partial yes | Partial yes | Yes | No | Yes | Yes | Yes | Yes | Yes | Yes | Critically low |
| Hui Wang,2020 | Yes | Yes | Yes | Partial yes | Yes | Yes | Partial yes | Partial yes | Yes | No | Yes | Yes | No | Yes | No | Yes | Critically low |
| Matteo Rota,2020 | Yes | No | Yes | Partial yes | Yes | Yes | Partial yes | Partial yes | No | No | Yes | Yes | Yes | Yes | No | Yes | Critically low |
| Zhiwei Wang,2012 | Yes | No | Yes | Partial yes | Yes | Yes | Partial yes | Partial yes | No | No | Yes | No | No | Yes | Yes | Yes | Critically low |
| Xin-Hai Yin,2016 | Yes | No | Yes | Partial yes | Yes | Yes | Yes | Yes | Yes | No | Yes | Yes | Yes | Yes | No | Yes | Critically low |
| Gabriele Nagel,2018 | Yes | No | Yes | Partial yes | Yes | Yes | Partial yes | Partial yes | No | No | Yes | Yes | Yes | Yes | No | Yes | Critically low |
