# Supplementary material for: Risk factors for gastric cancer: A comprehensive analysis of observational studies
Source: Front Public Health. 2023 Jan 4;10:892468. doi: 10.3389/fpubh.2022.892468 (PMC9845896; doi:10.3389/fpubh.2022.892468)
Supplement: Supplementary file 3 [file Table_3.DOCX]

**Supplementary Table3. Evaluation of the epidemiologic evidence quality.**

| **Risk/Protective factors** | **Exposure** | Exposure contrast | **Author,year** | **Precision of the estimate** | | **Consistency of results** | **No evidence of small-study effects** |
| --- | --- | --- | --- | --- | --- | --- | --- |
|  |  |  |  | > 1000 disease cases | P < 0.001 | (I2<50% and Cochran Q test P>0.10) | (P > 0.10) |
| **Associations supported by high evidence** | | |  |  |  |  |  |
| **Risk factors:** | Waist circumference | HvL | Xuan Du,2017 | Yes | Yes | Yes | Yes |
|  | Bacon | HvL | Hongcheng Zhu,2013 | Yes | Yes | Yes | Yes |
| **Protective factors:** | D-TAC | HvL | MohammadParohan,2019 | Yes | Yes | Yes | Yes |
|  | Vegetable fat | HvL | Jun Han,2015 | Yes | Yes | Yes | Yes |
|  | Cruciferous vegetable | HvL | Qi-Jun Wu,2013 | Yes | Yes | Yes | Yes |
|  | Cabbage | HvL | Qi-Jun Wu,2013 | Yes | Yes | Yes | Yes |
|  | Total Vitamin | HvL | Pengfei Kong,2014 | Yes | Yes | Yes | Yes |
|  | Vitamin A | 1.5 mg/d increment | Pengfei Kong,2014 | Yes | Yes | Yes | Yes |
|  | Vitamin C | 100 mg/d increment | Pengfei Kong,2014 | Yes | Yes | Yes | Yes |
|  | Years of fertility | HvL | M Constanza Camargo,2012 | Yes | Yes | Yes | Yes |
| **Associations supported by moderate evidence** | |  |  |  |  |  |  |
| **Risk factors:** | Refined grain | HvL | Tonghua Wang,2020 | Yes | Yes | No | Yes |
|  | Red meat | 100 g/d increment | Seong Rae Kim,2019 | Yes | Yes | No | Yes |
|  | Processed meat | HvL | Seong Rae Kim,2019 | Yes | Yes | No | Yes |
|  | Chili | M-HvL | Yanbin Du,2020 | Yes | Yes | No | Yes |
|  | Total alcohol | HvL | Peng-Liang Wang,2017 | Yes | Yes | No | Yes |
|  | PPI | regular vs. not | Ruijie Zeng,2021 | Yes | Yes | No | Yes |
|  | Smoking | ever vs. never | Ana Ferro,2018 | Yes | Yes | No | Yes |
|  | Depression | present vs. absent | Yuehua Zhang,2021 | Yes | Yes | No | Yes |
|  | Pernicious anemia | present vs. absent | Minkyo Song,2019 | Yes | Yes | No | Yes |
|  | Diabetes mellitus,type 1 | present vs. absent | Minkyo Song,2019 | No | Yes | Yes | Yes |
|  | HP | present vs. absent | Yoon Park,2021 | Yes | Yes | No | Yes |
|  | EBV | present vs. absent | Jong-Myon Bae,2016 | Yes | Yes | No | Yes |
|  | ABO blood group | blood group A vs non-A | Zhiwei Wang,2012 | Yes | Yes | No | Yes |
| **Protective factors:** | Mediterranean diet score | HvL | Jakub Morze,2021 | Yes | Yes | No | Yes |
|  | Fiber | HvL | Zhizhong Zhang,2013 | Yes | Yes | No | Yes |
|  | Allium vegetable | HvL | Yong Zhou,2011 | Yes | Yes | No | Yes |
|  | Garlic | HvL | Federica Turati,2015 | Yes | Yes | No | Yes |
|  | Carrot | HvL | Hossein Fallahzadeh,2015 | Yes | Yes | Yes | No |
|  | Vitamin C | HvL | Peiwei Li,2014 | Yes | Yes | No | Yes |
|  | Vitamin E | 10 mg/d increment | Pengfei Kong,2014 | Yes | Yes | No | Yes |
|  | β-carotene | HvL | Peiwei Li,2014 | Yes | Yes | No | Yes |
|  | Total Polyphenols | HvL | Facundo Vitelli-Storelli,2020 | Yes | Yes | No | Yes |
|  | Aspirin | regular vs. not | C Bosetti，2020 | Yes | Yes | No | Yes |
|  | Physical activity | HvL | Theodora Psaltopoulou,2016 | Yes | Yes | No | Yes |
|  | Refrigerator use | yes vs.no | Shijiao Yan,2018 | Yes | Yes | No | Yes |
|  | ABO blood group | blood group 0 vs non-0 | Zhiwei Wang,2012 | Yes | Yes | No | Yes |
| **Associations supported by weak evidence** | |  |  |  |  |  |  |
| **Risk factors:** | BMI | ≥30 vs 18.5-25 | Xue-Jun Li,2014 | Yes | No | Yes | Yes |
|  | Waist to hip ratio | HvL | Xuan Du,2017 | Yes | No | Yes | Yes |
|  | DII | HvL | Ying Liang,2019 | No | Yes | Yes | No |
|  | DII | per 1 unit | Ying Liang,2019 | No | No | No | Yes |
|  | Dietary cholesterol | HvL | Peng Miao,2021 | Yes | No | No | Yes |
|  | Saturated fat | HvL | Jun Han,2015 | Yes | No | No | Yes |
|  | Total meat | HvL | Ana Ferro,2019 | Yes | No | No | Yes |
|  | Red meat | HvL | Seong Rae Kim,2019 | Yes | Yes | No | No |
|  | Beef | HvL | Hongcheng Zhu,2013 | Yes | No | Yes | Yes |
|  | Processed meat | 50 g/d increment | Seong Rae Kim,2019 | Yes | Yes | No | No |
|  | Sausage | HvL | Hongcheng Zhu,2013 | Yes | No | No | No |
|  | Salt | HvL | Sheng Ge,2012 | Yes | Yes | No | No |
|  | Pickled foods | HvL | Lanfranco D'Elia,2012 | Yes | No | Yes | No |
|  | Salted fish | HvL | Lanfranco D'Elia,2012 | Yes | No | Yes | Yes |
|  | Nitrite intake | HvL | Fei-Xiong Zhang,2019 | Yes | No | No | No |
|  | Nitrite intake | MvL | Fei-Xiong Zhang,2019 | Yes | No | No | Yes |
|  | Nitrosamines | HvL | Peng Song,2015 | NA | No | No | Yes |
|  | Fermented soybean products | HvL | Yameng Wang,2021 | Yes | No | No | Yes |
|  | Total alcohol | 12.5 g/d increment | Peng-Liang Wang,2017 | Yes | No | No | Yes |
|  | Beer | HvL | Peng-Liang Wang,2017 | Yes | No | Yes | No |
|  | Beer | 12.5 g/d increment | Peng-Liang Wang,2017 | Yes | No | Yes | Yes |
|  | Liquor | HvL | Peng-Liang Wang,2017 | Yes | No | No | Yes |
|  | Smoking | current vs.never | Ricardo Ladeiras-Lopes,2008 | NA | Yes | No | Yes |
|  | Smoking | former vs.never | Ricardo Ladeiras-Lopes,2009 | NA | Yes | No | Yes |
|  | NAFLD | present vs. absent | Shou-Sheng Liu,2020 | No | No | No | No |
|  | SLE | present vs. absent | Ann EClarkeMD,2021 | No | No | Yes | Yes |
|  | Diabetes mellitus | present vs. absent | T Tian,2012 | Yes | No | No | Yes |
|  | GDM | present vs. absent | Y Wang,2020 | No | No | Yes | No |
|  | HBV | present vs. absent | Yusha Yang,2021 | NA | No | No | Yes |
|  | HCV | present vs. absent | Yusha Yang,2021 | NA | No | No | Yes |
|  | HCMV | present vs. absent | Hui Wang,2020 | No | No | No | No |
|  | HPV | present vs. absent | Hui Wang,2020 | No | No | No | Yes |
|  | JCV | present vs. absent | Hui Wang,2020 | No | No | No | Yes |
|  | Tooth Loss | HvL | Xin-Hai Yin,2016 | Yes | No | No | Yes |
| **Protective factors:** | Fiber | 10 g/d increment | Zhizhong Zhang,2013 | No | Yes | Yes | NA |
|  | Whole grain | HvL | Xiao-Feng Zhang,2020 | Yes | Yes | No | No |
|  | Polyunsaturated fat | HvL | Jun Han,2015 | Yes | No | No | Yes |
|  | White meat | HvL | Seong Rae Kim,2019 | Yes | No | No | Yes |
|  | Nitrate intake | HvL | Fei-Xiong Zhang,2019 | Yes | No | No | No |
|  | Nitrate intake | MvL | Fei-Xiong Zhang,2019 | Yes | No | No | Yes |
|  | Fruit | HvL | Qingbing Wang,2014 | Yes | No | Yes | Yes |
|  | Fruit | 100 g/d increment | Qingbing Wang,2014 | Yes | No | No | Yes |
|  | Citrus fruit | HvL | Paola Bertuccio,2019 | Yes | No | No | Yes |
|  | Onion | HvL | Federica Turati,2015 | Yes | Yes | No | No |
|  | Tomato | HvL | Tingsong Yang, 2013 | Yes | No | No | Yes |
|  | Nuts | HvL | Dai Zhang,2020 | Yes | No | No | Yes |
|  | Total soy products | HvL | Yameng Wang,2021 | Yes | Yes | No | No |
|  | Non-fermented soybean products | HvL | Yameng Wang,2021 | Yes | Yes | No | No |
|  | Vitamin A | HvL | YihuaWu,2015 | Yes | No | No | Yes |
|  | Vitamin E | HvL | Peiwei Li,2014 | Yes | Yes | No | No |
|  | α-carotene | HvL | Peiwei Li,2014 | Yes | No | No | Yes |
|  | Aspirin | ≥5 yrs vs. not | Lijuan Wang,2021 | Yes | No | No | Yes |
|  | Statins | ever vs.never | P P Singh,2013 | Yes | No | No | Yes |
|  | Menopausal hormone therapy | ever vs.never | M Constanza Camargo,2012 | Yes | No | Yes | Yes |
|  | Toothbrushing frequency | HvL | Huadong Wu,2021 | Yes | No | Yes | Yes |
|  | Education level | HvL | Matteo Rota,2020 | Yes | No | No | Yes |
|  | Household income | HvL | Matteo Rota,2020 | No | No | No | Yes |
| **Non-significant associations (P＞0.05)** | |  |  |  |  |  |  |
|  | Height | per 5cm | Min Seok Seo,2020 | Yes | No | No | Yes |
|  | BMI | 25-30 vs 18.5-25 | Xue-Jun Lin,2014 | Yes | No | No | No |
|  | Glycemic index (GI) | HvL | Federica Turati,2019 | Yes | No | No | Yes |
|  | Glycemic load (GL) | HvL | Federica Turati,2019 | Yes | No | No | Yes |
|  | Carbohydrate | HvL | Yao Ye,2017 | NA | No | No | Yes |
|  | Dietary cholesterol | 100 mg/d increment | Peng Miao,2021 | Yes | No | No | Yes |
|  | Total fat | HvL | Jun Han,2015 | Yes | No | No | Yes |
|  | Monounsaturated fat | HvL | Jun Han,2015 | Yes | No | No | Yes |
|  | Animal fat | HvL | Jun Han,2015 | Yes | No | Yes | Yes |
|  | Pork | HvL | Hongcheng Zhu,2013 | Yes | No | Yes | Yes |
|  | Ham | HvL | Hongcheng Zhu,2013 | Yes | No | No | No |
|  | White meat | 100 g/d increment | Seong Rae Kim,2019 | Yes | No | No | No |
|  | Fish | HvL | Shengjun Wu,2011 | Yes | No | No | No |
|  | Miso-soup | HvL | Lanfranco D'Elia,2012 | Yes | No | Yes | Yes |
|  | Vegetable | HvL | Qingbing Wang,2014 | Yes | No | Yes | Yes |
|  | Vegetable | 100 g/d increment | Qingbing Wang,2014 | Yes | No | No | No |
|  | Liquor | 12.5 g/d increment | Peng-Liang Wang,2017 | Yes | No | Yes | Yes |
|  | Wine | HvL | Peng-Liang Wang,2017 | Yes | No | No | Yes |
|  | Wine | 12.5 g/d increment | Peng-Liang Wang,2017 | Yes | No | No | No |
|  | Tea | HvL | Long-Gang Zhao,2021 | Yes | No | No | Yes |
|  | Tea | 1 cup/d increment | Long-Gang Zhao,2021 | Yes | No | Yes | Yes |
|  | Black tea | HvL | Long-Gang Zhao,2021 | Yes | No | Yes | Yes |
|  | Black tea | 1 cup/d increment | Long-Gang Zhao,2021 | Yes | No | Yes | Yes |
|  | Green tea | HvL | Yanhong Huang,2017 | Yes | No | No | Yes |
|  | Green tea | 1 cup/d increment | Long-Gang Zhao,2021 | Yes | No | No | Yes |
|  | Coffee | HvL | Feiyue Xie,2014 | Yes | No | No | No |
|  | Sugar-sweetened beverages(SSB) | HvL | Yuting Li,2021 | Yes | No | No | No |
|  | Dairy product | HvL | Yan Sun,2014 | Yes | No | No | Yes |
|  | Milk | HvL | Yan Sun,2014 | NA | No | No | Yes |
|  | Cheese | HvL | Yan Sun,2014 | NA | No | Yes | Yes |
|  | Retinol | HvL | YihuaWu,2015 | Yes | No | No | Yes |
|  | Vitamin D | HvL | Saeid Khayatzadeh,2015 | Yes | No | Yes | Yes |
|  | Folate | HvL | Martin Tio,2014 | Yes | No | No | Yes |
|  | Selenium | HvL | Marco Vinceti,2018 | No | No | No | Yes |
|  | Zinc | HvL | Peiwei Li,2014 | Yes | No | No | Yes |
|  | Isoflavones | HvL | Jie You,2018 | Yes | No | Yes | Yes |
|  | Flavonoid | HvL | Yacong Bo,2016 | Yes | No | No | Yes |
|  | Anthocyanins | HvL | DeYi Yang，2020 | Yes | No | Yes | Yes |
|  | Metformin | ever vs.never | Y.Shuai,2020 | NA | No | No | Yes |
|  | Bisphosphonates | ever vs.never | Ellen Wright,2015 | Yes | No | Yes | Yes |
|  | Sedentary Behavior | HvL | Daniela Schmid,2014 | Yes | No | Yes | Yes |
|  | IBD | present vs. absent | Qianyi Wan,2021 | NA | No | Yes | Yes |
|  | HTLV-1 | present vs. absent | Hui Wang,2020 | No | No | Yes | NA |
|  | PM2.5 | Per 5 μg/m3 increase | Gabriele Nagel,2018 | No | No | Yes | No |
